# Does Extended Interval Dosing Natalizumab Preserve Effectiveness in Multiple Sclerosis? A 7 Year-Retrospective Observational Study

**DOI:** 10.3389/fimmu.2021.614715

**Published:** 2021-03-25

**Authors:** Javier Riancho, Sonia Setien, Jose Ramón Sánchez de la Torre, Marta Torres-Barquin, Mercedes Misiego, José Luis Pérez, Tamara Castillo-Triviño, Cristina Menéndez-García, Manuel Delgado-Alvarado

**Affiliations:** ^1^ Service of Neurology, Hospital Sierrallana-IDIVAL, Torrelavega, Spain; ^2^ Department of Medicine and Psychiatry, University of Cantabria, Santander, Spain; ^3^ Centro de Investigación en Red de Enfermedades Neurodegenerativas, CIBERNED, Instituto Carlos III, Madrid, Spain; ^4^ Red Española de Esclerosis Múltiple, Madrid, Spain; ^5^ Service of Radiology, Hospital Sierrallana, Torrelavega, Spain; ^6^ Service of Neurology, Hospital Universitario Donostia, San Sebastian, Spain; ^7^ Biodonostia Health Research Institute, San Sebastian, Spain; ^8^ Biomedical Research Networking Center for Mental Health (CIBERSAM), Madrid, Spain

**Keywords:** extended interval dosing, multiple sclerosis, natalizumab, disease modifying therapy, treatment

## Abstract

The extended interval dosing (EID) of natalizumab has been suggested to be associated with a reduced risk of progressive multifocal leukoencephalopathy (PML) and short-term preservation of efficacy but its long-term effectiveness remain unknown. We aimed to determine the long-term effectiveness and safety of natalizumab in an EID setting in a cohort of patients with multiple sclerosis (MS) treated for more than 7 years. We conducted an observational retrospective cohort study, including 39 (34 female, 5 male) patients with clinically definite relapsing-MS, initially treated with standard interval dosing (SID) of natalizumab (mean time 54 months [SD29]) who were then switched to EID, every 8 weeks (mean time 76 months [SD13]). The main outcome measures included the following: i) annualized relapse rate (ARR), ii) radiological activity, iii) disability progression, and iv) NEDA-3 no evidence of disease activity index. EID preserved ARR, radiological activity, and prevented disability worsening during follow-up. The proportion of patients maintaining their NEDA-3 status after 24, 48, and 72 months of natalizumab administration in EID was 94%, 73%, and 70%, respectively. Stratified analysis according to history of drug therapy showed that the EID of natalizumab was slightly more effective in naïve patients than in those previously treated with other immunosuppressive drugs. No cases of PML or other severe adverse reactions were reported. In conclusion, long-term therapy with natalizumab in an EID setting following an SID regimen maintained its disease-modifying activity, and was safe and well tolerated for over 7 years. These encouraging observational results need to be confirmed in controlled clinical trials.

## Introduction

The humanized monoclonal antibody natalizumab (Tysabri^®^; Biogen-Idec, Cambridge, MA, USA) is directed against the α4 subunit of both α4β1 and α4β7 integrins. The blockage of these integrins, which are expressed on the cellular surface of circulating mononuclear cells, prevents their entry into the central nervous system (CNS) through the blood-brain barrier (1). Natalizumab administered every 4 weeks reduces CNS inflammation, and thus it is a rapidly-acting and effective agent in reducing both clinical and radiological activity, as well as preventing disability progression in patients with multiple sclerosis (MS) (2, 3). As a consequence of its mechanism of action, natalizumab, albeit usually well tolerated, has been associated with an increased risk of progressive multifocal leukoencephalopathy (PML), a rare life-threatening infection caused by the John Cunningham virus (JCV) (4, 5). Presently, it is widely accepted that the risk of PML is particularly high in patients who have previously received immunosuppressive drugs, in JCV positive patients (index > 1.5), and in those treated with natalizumab for more than 24 months (5). In contrast, the discontinuation of natalizumab has been associated with MS reactivation and rebound (6). In this scenario, clinicians treating patients with MS who are at a high risk of PML must carefully consider either continuing treatment with natalizumab or switching to another highly-effective therapy (6, 7). For patients receiving long-term natalizumab treatment, several therapeutic strategies have been suggested to reduce the risk of PML. Among them, several investigators have suggested extended interval dosing (EID) schedules, most of them involving drug administration every 6 to 8 weeks (8–11). EID seems to result in a partial desaturation of drug receptors that might allow restoring some degree of anti-viral immune response (1, 12). On this basis, the natalizumab product information sheet has been recently amended to include the possibility of using EID (with dosing every 6 weeks) in patients at high risk of PML (https://www.ema.europa.eu/en/documents/product-information/tysabri-epar-product-information_en.pdf). Moreover, a few studies suggest that treatment with EID of natalizumab is associated with a lower PML risk, while preserving the effectiveness on the control of disease activity (8–11). However, these studies included small groups of patients who were followed-up for short periods (8–11). Confirmation of the effect of EID is critically important for clinicians to be able to discuss and help patients take informed decisions regarding a long-term therapeutic plan once the disease activity is controlled. Therefore, we aimed to analyze a quite unique cohort of patients with MS, followed-up for more than 7 years, to study the efficacy and safety of treatment with natalizumab in an EID setting.

## Patients and Methods

The present study was motivated by a recent organizational change at our hospital, in which one author (JR) was asked to take care of a cohort of patients with MS. This was an observational retrospective cohort study with analysis of data collected during routine clinical practice at Hospital Universitario Sierrallana, in Cantabria, Spain. The protocol was approved by the institutional review board [Comité de Ética de la Investigación con medicamentos de Cantabria (CEIm Cantabria), reference number: 2019.328] and the study was conducted in accordance to the relevant guidelines and regulations.

The inclusion criteria were as follows: i) a diagnosis of clinically definite relapsing-MS, according to the McDonald revised criteria (13); ii) age over 18 years; iii) previous treatment with SID of natalizumab (every 4 weeks) for at least 24 months; and iv) treatment switched to EID of natalizumab (every 8 weeks).

Clinical charts were reviewed to collect the following variables: sex, age at diagnosis, symptoms at onset, previous treatments, duration of treatment with natalizumab in SID, reason for natalizumab extension, duration of treatment with natalizumab in EID, clinical relapses during treatment, magnetic resonance imaging (MRI) lesion load, presence of gadolinium-enhanced lesions, and the Expanded Disability Status Scale (EDSS) score. In addition, we carefully checked for potential natalizumab-related adverse reactions, specifically PML. Serologic JCV status was monitored every 6 months.

The main outcome measures were as follows: i) the annualized relapse rate (ARR), ii) presence of brain MRI activity (considered as at least 2 new T2-hyperintense lesions and/or new gadolinium-enhancing lesions), iii) EDSS score, and iv) disability progression assessed by the EDSS and defined as an increase of 1.5, 1 or 0.5 points in patients with MS having a previous EDSS score of 0, < 5.5, and ≥ 5.5, respectively. As an outcome parameter of global disease control, we estimated the no evidence of disease activity (NEDA-3) status, which includes the combined absence of clinical relapses, radiological activity as well as disability progression.

In a complementary analysis, patients were stratified according to history of previous drug therapy. Thus, we divided patients into “switchers” if they had previously undertaken other disease modifying therapy (DMT) and “naïve” if natalizumab was the first DMT used.

Baseline characteristics were compared by the non-parametric Mann–Whitney U test and the Fisher exact test. Global differences in ARR and EDSS across groups were tested by the Kruskal-Wallis test. Subsequently, the Wilcoxon test was used for pairwise between-group comparisons. Kaplan-Meier analyses were used to assess the proportion of patients who maintained their NEDA-3 status and an EDSS score < 6. Differences were then tested by the Gehan-Breslow-Wilcoxon test. This procedure was also used to compare differences in the course of the NEDA-3 status between switchers and naïve patients. p-values < 0.05 were considered as significant. Prism software (GraphPad Software Inc., San Diego, California) was used for statistical analysis.

## Results

### Patients’ Characteristics

Thirty-nine patients (34 female and 5 male; mean age at diagnosis, 33 years) were included in the study. The patient characteristics have been summarized in **Table 1**. Among them, 26 patients had been previously treated with other DMTs (25, interferon; 1, azathioprine), while in 13 patients (33%) natalizumab had been chosen as the initial DMT. Regarding treatment with natalizumab, all patients included in the study followed the same therapeutic regimen; they were treated with natalizumab in an SID setting for at least 24 months. Subsequently, because of safety concerns, and after having evaluated other therapeutic options, the dosing schedule was switched to EID. The primary reason for extending the dosing interval of natalizumab was the concern of a high risk of PML. At the inception of this cohort there were very scarce data. Therefore, it was opted for a potentially safer 8-week scheduling.

**Table 1:** Main patients characteristics.

Pre-Natalizumab		
Number of patients	39 33 (10.4) 34 (87%) 25 (64%) 1 (2.5%) 0.45 (0.53)	
Age at diagnosis, mean (SD)		
Females, n (%)		
Previous DMTs -IFNβ, n (%) -AZA, n (%)		
Pre-Natalizumab AAR (patients treated with DMTs)		
**Natalizumab**		
	**SID (4 weeks) (n=39)**	**EID (8 weeks) (n=39)**
Age at the beginning, mean (SD)	38.97 (11.10)	43.41 (10.71)
Duration of treatment, mean (SD)	51.12 months (19.89)	76.68 months (13.31)
JCV +, n(%)	–	32 (82%)
EDSS at the beginning, median [IR]	2 [1-3.5]	2 [1-3.5]
ARR, mean (SD)	0.03 (0.09)	0.02 (0.06)
Radiological activity	0.05 (0.03)	0.04 (0.03)
EDSS at the end of the treatment, median [IR]	2 [1-3.5]	2 [1-3.5]
Adverse reactions (clinical) (n,[%])	Respiratory infection (5 [13%]) Urinary infection (4 [10%]) Pharyingitis (3 [8%]) Diarrhea (1 [3%]) Herpes labialis (1[3%]) Headache (1[3%])	Urinary infection (6 [15%]) Respiratory infection (2 [5%]) Pharyingitis (2 [5%]) Pneumonia (1[3%]) Diarrhea (1[3%]) Herpes labialis (1[3%]) Herpes zoster (1[3%]) External otitis (1[3%])
Adverse reaction (analytical) (n,[%])	Mild lymphocytosis (27 [70%]) Mild liver test alteration (4 [10%]) Mild granulocytosis (3 [8%]) Decreased mean platelet volume (3 [8%]) Anemia (1 [3%])	Mild lymphocytosis (21 [54%]) Mild granulocytosis (2 [5%]) Decreased mean platelet volume (2 [5%]) Anemia (1 [3%])

ARR, annualized relapse rate; AZA, azathioprine; DMT, disease modifying therapies; EID, expanded interval dosing; EDSS, expanded disability status scale; IFNβ, interferon beta; IR, interquartile range; JCV, John Cunningham virus; SD, standard deviation; SID, standard interval dosing. Radiological activity was defined as the appearance of at least 2 new T2-hyperintense lesions and/or new gadolinium-enhancing lesions.

In this context, at the initiation of EID of natalizumab, 32 out of 39 patients (82%) were seropositive for JCV (quantitative data concerning the evolution of the JCV index was not available for all patients). Of note, at the completion of this study, the JCV index was low (<0.9) in 6 patients, intermediate (0.9–1.5) in 4, and high (>1.5) in 22 patients. The mean age at the SID initiation of natalizumab was 39 years (SD, 11) and mean duration of treatment with SID of natalizumab was 51 months (SD, 20).

Regarding the EID of natalizumab, patients’ mean age at initiation was 43 years (SD, 10) and the mean duration of treatment was 77 months (SD, 13).

Natalizumab administration in both, SID and EID regimens, was well tolerated. We did not find any case of PML or any other severe adverse reactions leading to natalizumab discontinuation during the administration of SID or EID regimens (**Table 1**). The most frequent adverse effects were respiratory and urinary tract infections.

### ARR, Radiological Activity, and Disability Progression

**Figure 1:**
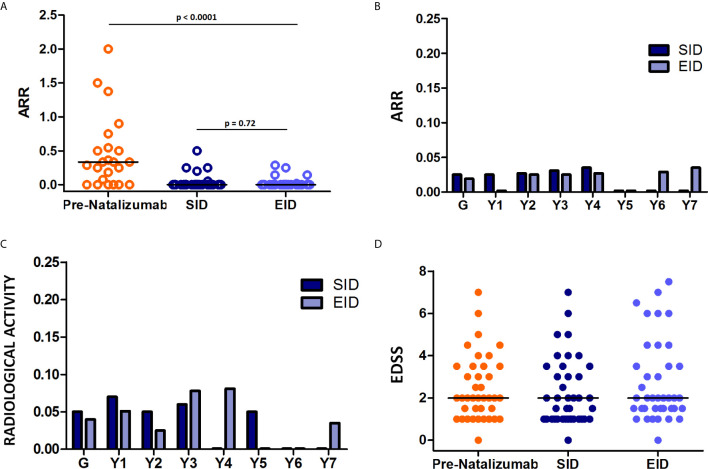
Annualized relapse rate (ARR) and disability progression in patients treated with natalizumab in extended interval dosing (EID). **(A)** AAR before natalizumab treatment (Pre-Natalizumab, orange), during the standard interval dosing (SID, dark blue), and EID (light blue) of natalizumab. A significant difference was found between the studied groups (Kruskal-Wallis test, p<0.0001). ARR did not significantly vary between the SID and EID groups (Wilcoxon test, p=0.72). **(B)** Detailed ARR during the 7-year follow-up of patients treated with natalizumab in SID (dark blue) and EID (light blue). Y1-Y7: ARR during years 1 through 7 in patients on natalizumab in SID and EID. **(C)** Radiological activity during the follow-up of patients treated with natalizumab in SID (dark blue) and EID (light blue). Y1-Y7: radiological activity during years 1 through 7 in patients on natalizumab in SID and EID. Radiological activity was defined as the appearance of at least 2 new T2-hyperintense lesions and/or new gadolinium-enhancing lesions. **(D)** Expanded Disability Status Scale (EDSS) score before natalizumab treatment (pre-natalizumab, orange), during treatment with natalizumab in SID (dark blue) and EID (light blue) settings. No significant differences were noted among the three groups (Kruskal-Wallis test, p=0.46).

Regarding the ARR, a significant difference was found between the study groups (p<0.0001) (**Figure 1A**). After initiating treatment with SID of natalizumab, the ARR significantly decreased from 0.54 (SD, 0.60) to 0.03 (SD, 0.09; p=0.0005) (**Figure 1A**). However, the ARR did not vary significantly between the SID and EID groups (SID-ARR, 0.025 [SD, 0.026]; EID-ARR, 0.02 [SD, 0.06]; p 0.72) (**Figure 1A**). Specifically, ARR remained low during the entire period of treatment with natalizumab in both SID and EID regimens, ranging from 0 to 0.036 and 0 to 0.035, respectively throughout the 7-year follow-up period (**Figure 1B**).

The radiological activity also remained low in both groups of patients with MS receiving the two natalizumab regimens throughout the follow-up period (SID, 0.050 [SD, 0.03]; EID, 0.040 [SD, 0.03]; p= 0.67). Specifically, it ranged from 0 to 0.076 and 0 to 0.081 in the SID and EID groups, respectively (**Figure 1C**). Analysis of ARR and radiological activity showed some discrepancies, and ARR did not always correlate well with radiological activity (for example, in year 1 of the EID regimen, radiological activity was relatively high whereas ARR remained very low). Of note, ARR represented clinical relapses alone and not radiological activity (**Figures 1B, C**). Concerning disability progression, no significant variations in EDSS scores were observed during the follow-up period (Pre-Natalizumab: median, 2; [interquartile range (IQ), 1–3.5]; Natalizumab-SID: median, 2; IQ, 1–3.5; Natalizumab-EID: median, 2; IQ, 1.5–3.5; p=0.46) (**Figure 1D**).

**Figure 2:**
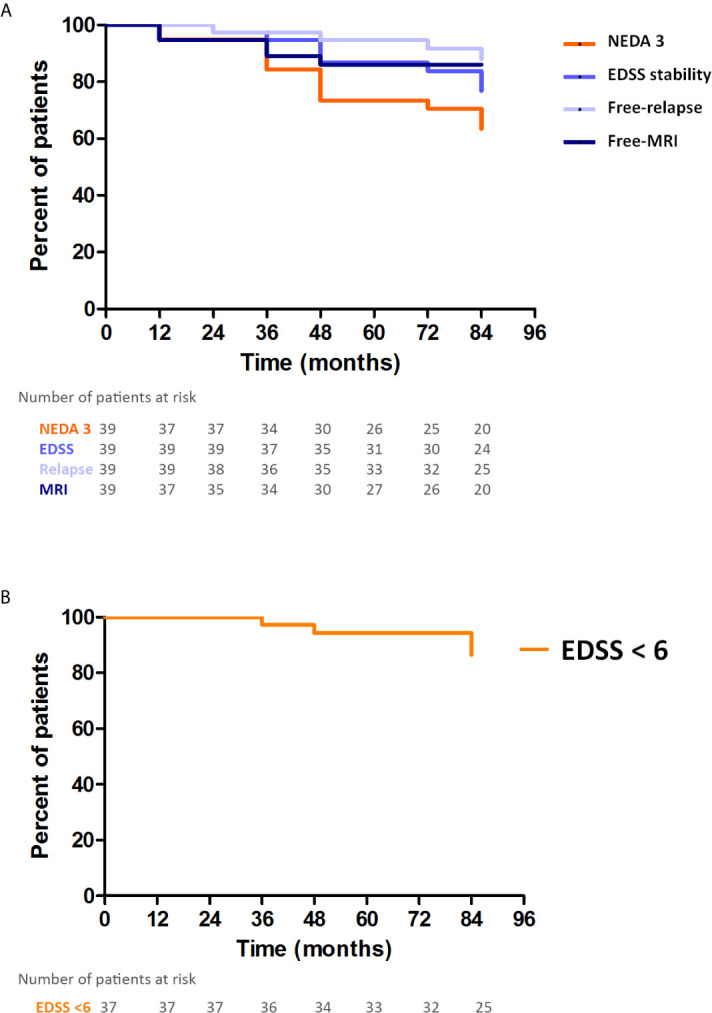
Preservation of the no evidence of disease activity (NEDA-3) status and Expanded Disability Status Scale (EDSS) score < 6 in patients treated with natalizumab in extended interval dosing (EID). **(A)** Kaplan-Meyer plots representing the proportion of patients i) maintaining the NEDA-3 status (orange), ii) showing no worsening of the EDSS score (light blue), iii) showing no evidence of clinical relapse (purple), and iv) showing no evidence of radiological activity while on an EID of natalizumab (dark blue). **(B)** Kaplan-Meyer plot of the proportion of patients maintaining an EDSS score < 6 while on an EID of natalizumab.

The beneficial effect of natalizumab-EID in maintaining functional status was confirmed by the Kaplan-Meier analysis. As shown in **Figure 2A**, the proportion of patients maintaining NEDA-3 status was 94%, 73%, and 70% after 24, 48, and 72 months of therapy with EID regimen, respectively. At month 72 of the EID regimen, 83% of patients showed no disability progression and 86% showed no clinical relapses (**Figure 2A**). In addition, after 84 months of treatment with EID regimen, more than 85% of patients maintained an EDSS score < 6 (**Figure 2B**).

### Natalizumab-EID in Switchers and Naïve Patients

**Figure 3:**
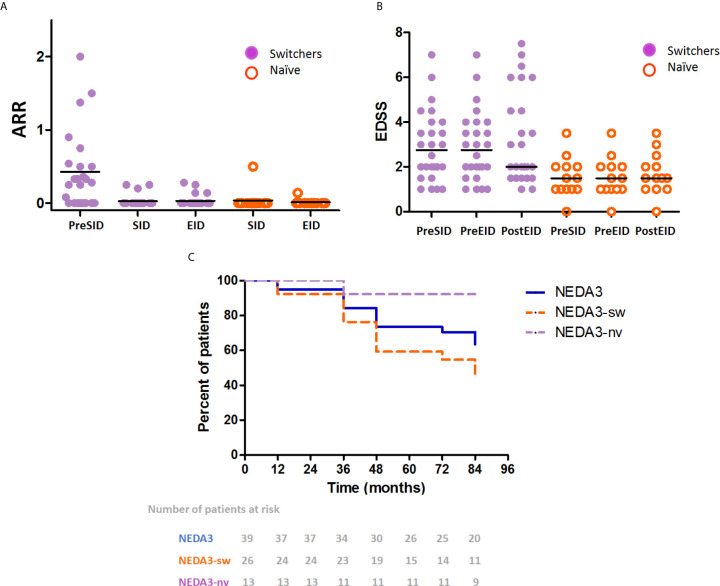
Extended interval dosing (EID) of natalizumab in switchers and naïve patients. **(A)** The mean annualized relapse rate (AAR) before natalizumab treatment (Pre-SID), during treatment with natalizumab in standard interval dosing (SID), and in EID in switchers (purple) and naïve (orange) patients. A significant decrease was evidenced in switchers after initiating treatment with natalizumab (Wilcoxon test, p=0.0008). ARR remained low in both naïve patients and switchers treated with natalizumab in SID and EID. **(B)** The median Expanded Disability Status Scale (EDSS) scores before natalizumab treatment (Pre-SID), before EID (Pre-EID), and at the end of EID period (Post-EID) in switchers (purple) and naïve (orange) patients. Although switchers exhibited a significantly higher EDSS score, the score remained stable all through the follow-up period in both groups. **(C)** Kaplan-Meyer plots of the proportion of patients maintaining the no evidence of disease activity (NEDA-3) status (global data: blue line; switchers: orange dashed line; naïve: purple dashed line; Gehan-Breslow Wilcoxon test p=0.012).

In a complementary analysis, patients were divided into two groups depending on whether they had been treated with other DMTs prior to natalizumab-SID (“switchers”) or not (“naïve”). The cohort included 26 switchers and 13 naïve patients. No sex differences were evidenced between groups (switchers: female, 22; males 4; naïve: female, 12; male, 1; p=0.45). Of note, switcher patients were slightly older than naïve patients (mean age, 41 *vs.* 34 years; p=0.05), and exhibited a more advanced disease status (mean EDSS score, 2.75 *vs.* 1.50; p=0.006). No significant differences were observed in the mean duration of treatment with the SID regimen (switchers *vs.* naïve: 39 *vs.* 38 months; p=0.11) and EID regimen (switchers *vs*. naïve: 76 *vs*. 78 months; p=0.24) between groups. Primary patient data are summarized in (**Table 2**). Among switchers, ARR significantly decreased after initiating SID of natalizumab (from 0.42 [SD, 0.53] to 0.026 [SD, 0.07]; p=0.0008) and remained at the same level when these patients were treated with the EID regimen (p > 0.99) (**Figure 3A**). In naïve patients, the ARR remained low with both SID (0.038 [SD 0.13]) and EID (0.010 [SD 0.03]) regimens, without significant differences between the two periods (p > 0.99) (**Figure 3A**). In concern to radiological activity, no significant differences were found after extending natalizumab administration from SID to EID in both switchers and naïve patients (switchers: 0.05 [0.04] vs 0.04 [0.04] p=0.94; naïve patients: 0.06 [0.05] vs 0.03 [0.04]; p = 0.20).

**Table 2:** EID natalizumab in switchers/naïve patients.

	Switchers n=26	Naïve n=13	p
**Gender**	22F, 4M	12F, 1M	0.45
**JCV +,n(%)**	22 (85%)	10 (77%)	0.66
**SID**			
**Age, mean (SD)**	41 (12)	34 (7)	**0.050**
**Duration, mean (SD)**	39 (11)	39 (18)	0.109
**EDSS, median [IR]**	2.75 [1.875-4]	1.5 [1-2]	**0.006**
**ARR, mean, (SD)**	0.026 (0.07)	0.038 (0.13)	0.790
**Radiological activity (SD)**	0.05 (0.04)	0.06 (0.05)	0.92
**EID**			
**Age, mean (SD)**	46 (11)	38 (7)	**0.020**
**Duration, mean (SD)**	76 (16)	78 (6)	0.240
**EDSS, median [IR]**	2.75 [1.875-4]	1.5 [1-2]	**0.006**
**ARR, mean, (SD)**	0.031 (0.07)	0.010 (0.03)	0.480
**Radiological activity (SD)**	0.04 (0.04)	0.03 (0.04)	0.286

Main patient’s characteristics. ARR, annualised relapse rate; EID, expanded interval dosing; EDSS, expanded disability status scale; IR, interquartile range; JCV, John Cunningham virus; SD, standard deviation; SID, standard interval dosing.

Regarding disability progression, although the baseline EDSS score at initiation of EID regimen was worse in switcher patients than in the naïve ones, the EDSS score was uniformly maintained during natalizumab-EID in both groups. In fact, among switchers, the median EDSS score was 2.75 pre-SID, 2.75 pre-EID, and 2 post-EID. Among the naïve patients, the EDSS score was maintained at 1.5 all through the three study time-points (**Figure 3B**).

Kaplan-Meier plots of NEDA-3 showed that naïve patients had a significantly more favorable control of disease activity, when compared to switchers (p=0.012). In this context, after 72 months of EID regimen 84 and 54% of naïve and switcher patients, respectively, maintained the NEDA-3 status (**Figure 3C**).

## Discussion

This study was conceived as an opportunity to assess the efficacy of administering natalizumab in an EID setting following a SID regimen in patients with MS who were at a high risk of PML. Monthly natalizumab is a highly effective regimen for the treatment of patients with MS (2, 3, 14). However, its long-term use is limited by an increased risk of PML, which is particularly high in patients seropositive for JCV, those previously treated with other immunosuppressant drugs, and in those receiving natalizumab for more than 2 years (5, 15–17). Based on its pharmacokinetics, it has been proposed that natalizumab in an EID setting might be associated with a lower risk of PML. Interestingly, cases of PML in patients with MS treated with natalizumab in an EID setting exhibit less severe disease course, characterized by a prolonged pre-symptomatic phase, pauci-symptomatic onset, low JCV load, less severe functional impairment during immune reconstitution, and a mild disability burden (18).

This is supported by several preclinical studies that reported that extending the dosing interval to 6–8 weeks resulted in a partial drug receptor desaturation, allowing a small proportion of lymphocytes to pass through the blood-brain-barrier, leading to some degree of viral protection (1, 12, 19).

However, there are no studies on the effectiveness of long-term EID regimen yet. The present study shows that a long-term EID regimen (up to 7 years) following an SID regimen exhibited a high effectiveness in controlling disease activity, as evidenced by parameters such as ARR, radiological activity, and disability progression. Although several previous studies involved larger sample size, these included patients with variable dosing intervals, ranging from 5 to 8 weeks (10, 11), thus complicating the analysis of effectiveness (8–11). In our study, all patients followed the same 8-week dosing schedule, which was well tolerated and safe, specifically concerning the risk of PML throughout the 7-year follow-up. Thus, our long-term results provide further support for natalizumab therapy in an EID setting, as suggested previously by a few studies with shorter follow-up (8–11). As expected, treatment with natalizumab in both SID and EID settings reduced both the clinical relapse rate and radiological activity. However, there were some discrepancies between ARR and radiological activity. At some time points of the EID period, there were no clinical relapses, despite some evidence of radiological activity, while at other time points, ARR was slightly higher than radiological activity. This has been described as the clinico-radiological paradox (20). In fact, MRI may be more sensitive than clinical observation to detect some mild (subclinical) relapses. It has been suggested that this may be explained, at least in part, to cortical plasticity (21). Thus, it might be speculated that EID regimens might protect more profoundly from clinically evident inflammatory activity than from subclinical radiological flares. However, our data cannot give a clear answer and further randomized trials are needed to either confirm or disprove this contention.

Stratification of patients according to previous use of other DMTs showed that natalizumab-EID had a beneficial effect on both switchers and naïve patients, maintaining ARR at low levels and limiting disability progression as assessed by the EDSS scores. In contrast to that observed with ARR, disability analysis among switcher patients revealed that the EDSS scores did not decrease after initiating natalizumab administration in an SID setting, but decreased slightly after treatment with the EID regimen. We do not have a clear explanation for the lack of disability improvement among switchers after switching to SID of natalizumab, as has been commonly reported in routine clinical practice (14, 22). Intriguingly, the proportion of patients maintaining the NEDA-3 status was slightly higher among naïve patients than among switchers. This could be related to the fact that patients in the latter group were initiated on treatment with natalizumab-SID at an advanced age and with a more advanced disease status than naïve patients. We speculate that treatment with natalizumab at earlier stages of the disease, in a more severe inflammatory state, might exert a more pronounced immunomodulatory effect that possibly delays long-term disease progression (22, 23). Nevertheless, considering the small sample size of our study, the results of the subgroup analysis should be interpreted cautiously.

To the best of our knowledge, this is the first study reporting the long-term effects of treatment with natalizumab in an EID regimen following an SID regimen. Importantly, the present study has some limitations due to its observational approach, lack of a comparison control group, and limited sample size. Regarding the last concern, the small sample size impeded further subgroup analyses. Therefore, these encouraging results await to be confirmed by ongoing clinical trials (https://clinicaltrials.gov/ct2/show/NCT03689972). Pending the completion of these trials, our findings provide useful information on efficacy and safety that help decision making by clinicians and patients confronting therapeutic options after several years of therapy with SID of natalizumab.

In conclusion, the present study provides new real-world evidence that long-term administration of natalizumab in an EID setting with an 8-week dosing interval following an SID regimen is safe and maintains therapeutic efficacy in MS. Clinical trials are needed to confirm the benefits of this therapeutic regimen.

## Data Availability Statement

The raw data supporting the conclusions of this article will be made available by the authors, upon reasonable request.

## Ethics Statement

The studies involving human participants were reviewed and approved by Comité de Ética de la Investigación con medicamentos de Cantabria (CEIm Cantabria). The ethics committee waived the requirement of written informed consent for participation.

## Author Contributions

JR: conception, data collection, analysis, and writing. SS: data collection and revision. JS data collection and revision. MT-B: radiological analysis. MM: data collection and revision. JP data collection. TC-T: critical revision. CM-G: radiological analysis. MD-A: data collection, writing and revision. All authors contributed to the article and approved the submitted version.

## Funding

This study was supported by IDIVAL (NVAL 16/11).

## Conflict of Interest

JR has received travel grants or speaking fees from Merck, Sanofi-Genzyme, Roche, Biogen, and Novartis. JS has received travel grants from Merck. MM has received travel grants or speaking fees from Merck, Sanofi-Genzyme, and Biogen. TC-T has received speaking/consulting fees and/or travel funding from Bayer, Biogen, Merck, Novartis, Roche, Sanofi-Genzyme, and Teva. MD-A has received travel grants or speaking fees from Merck, Novartis, and Sanofi-Genzyme.

The remaining authors declare that the research was conducted in the absence of any commercial or financial relationships that could be construed as a potential conflict of interest.
